# The Interplay Between Inflammation and Stromal Components in Pancreatic Cancer

**DOI:** 10.3389/fimmu.2022.850093

**Published:** 2022-04-14

**Authors:** Ying Li, Jing Wang, Haiyan Wang, Shaoqiang Zhang, Yingxin Wei, Shanglong Liu

**Affiliations:** ^1^ Department of Blood Transfusion, The Affiliated Hospital of Qingdao University, Qingdao, China; ^2^ Department of Operating Room, The Affiliated Hospital of Qingdao University, Qingdao, China; ^3^ Department of General Surgery, Peking Union Medical College Hospital, Chinese Academy of Medical Science & Peking Union Medical College, Beijing, China; ^4^ Department of Gastrointestinal Surgery, The Affiliated Hospital of Qingdao University, Qingdao, China

**Keywords:** pancreatic cancer, tumor microenvironment, inflammation, immune response, crosstalk

## Abstract

Inflammation involves interactions between various immune cells, inflammatory cells, chemokines and cytokines in pancreatic cancer. Cancer cells as well as surrounding stromal and inflammatory cells establish an inflammatory tumor microenvironment (TME). Inflammation is closely associated with immunity. Meanwhile, immune cells are involved in both inflammation and immune response. Tumor-promoting inflammation and tumor-suppressive immunity are two main characteristics of the tumor microenvironment in pancreatic cancer. Yet, the mechanism of inflammation and immune response in pancreatic cancer development is still unclear due to the dual role of some cytokines and the complicated crosstalk between tumor and stromal components in TME. In this review, we outline the principal cytokines and stromal cells in the pancreatic TME that are involved in the tumor-promoting and immunosuppressive effects of inflammation, and discuss the interaction between inflammation and stromal components in pancreatic cancer progression. Moreover, the clinical approaches based on targeting TME in pancreatic cancer are also summarized. Defining the mechanisms of interplay between inflammation and stromal components will be essential for further development of anti-cancer therapies.

## Introduction

Pancreatic cancer is one of the most lethal malignancies, with a 5-year survival rate of less than 5% (1). The etiology of pancreatic cancer is not completely clear. Recent studies have shown that pancreatic cancer cells are surrounded by inflammatory microenvironment formed by stromal components (2). Increasing attention has been paid to the microenvironment, which is composed of many non-tumor cells, to elucidate its biological characteristics and explore potential targets for the development of new therapies.

Inflammation is closely related to the occurrence, development and prognosis of tumors. Tumor-associated inflammation is a key characteristic of malignant tumors. Cytokines such as interleukin (IL), chemokines and lymphokines regulate the inflammatory response (3). The tumor microenvironment (TME) is a key factor in tumor evolution. Immunity and inflammation are two critical characteristics of the pancreatic TME, but the relationship between them is complex and not completely clear (4). In this review, we discuss the principal cytokines and stromal cells in the pancreatic TME that are involved in the tumor-promoting and immunosuppressive effects of inflammation. Moreover, the clinical approaches based on targeting TME in pancreatic cancer are also summarized.

## Characteristics of the TME in Pancreatic Cancer

The TME comprises the interaction between tumor cells and the surrounding stromal components, which form a complex environment conducive to the malignant behavior of tumor cells. The TME of pancreatic cancer has specific characteristics: 1) a large number of tight matrix components, such as pancreatic stellate cells (PSCs), tumor-associated fibroblasts (cancer-associated fibroblasts, CAFs), collagen deposits, hyaluronic acid and extracellular matrix. A histopathological hallmark is a desmoplastic reaction to the tumor. The pancreatic TME is featured with excessive stroma which takes 90% content of the tumor, creating a mechanical barrier to limit immune cells infiltration; 2) various types of immunosuppressive cells including regulatory T (Treg) cells, myeloid-derived suppressor cells (MDSCs) and tumor-associated macrophages (TAMs), which establish an immunosuppressed TME. Pancreatic cancer is typically known as immunologically ‘cold’ tumor; and 3) a large number of cytokines or chemokine produced by both tumor and inflammatory cells, including pro-inflammatory IL-1, IL-6, IL-8, IL-17 and TNFα; the anti-inflammatory IL-10; and the dual-face cytokine transforming growth factor β (TGF-β) (5). Moreover, the pancreatic TME is also characterized by poor vascular perfusion, hypoxia and low pH. Tumors surrounded by the components of TME weaken anti-tumor immune response, maintain proliferation, escape apoptosis, and promote inflammatory environment and angiogenesis.

The inflammatory response is activated in response to damage or pathogen invasion. When innate immune system is activated, monocytes and neutrophils migrate to the damaged site and release the inflammatory mediator interleukin, tumor necrosis factor α (TNF-α) and prostaglandins, resulting in local vasodilation, increased permeability, leukocyte exudation and subsequent removal of pathogens or tissue repair (6). Inflammation results in the production of active oxides and induced tissue repair, but an excessive inflammatory reaction lead to tissue fibrosis, epithelial cell metaplasia and cell carcinogenesis. Chronic inflammation orchestrates the tumor-promoting microenvironment that is associated with tumorigenesis. The inflammatory microenvironment not only promotes the occurrence and development of tumors, but also participates in tumor immune tolerance (7). Tumor cells recruit inflammatory cells to tumors by producing various inflammatory factors such as growth factors, cytokines and chemokines and alter the functional status of inflammatory cells (8). Tumor cells also activate fibroblasts in tumor tissues to reshape the TME. Various inflammatory cells and activated fibroblasts that infiltrate in tumor tissue in turn change the metabolism and functional state of tumor cells. Inflammatory cytokines (such as TNF, IL-6, IL-11, IL-17 and IL-22) trigger signal cascades, directly or indirectly stimulate key transcription factors (APL, NF-κB, STAT3, Yap or Notch), regulate the cell cycle, control apoptosis, promote cell dedifferentiation and migration (9). Cancer immunoediting and tumor-promoting inflammation may co-exist in the pancreatic TME (10, 11). These mediators include activated proto-oncoproteins, tumor suppressors, chemokines, cytokines and downstream effectors. These mediators’ function in signal networks drives remodeling of the pancreatic TME.

Immunosuppressive cells in pancreatic TME mainly include tumor associated macrophages (TAMs), regulatory T cells (Tregs), myeloid-derived suppressor cells (MDSCs) and CAFs. Cytokines mainly include IL-10, TGF-β, IL-17, PD-1/PD-L1 and other immune checkpoint molecules and exosomes (12). The inflammatory microenvironment, immunosuppressive microenvironment and angiogenesis microenvironment together constitute a pancreatic TME with low oxygen, low pH and high pressure. Immune tolerance and inflammation are two main characteristics of the TME, but the specific relationship between them is not clear. The pancreatic TME shows abnormal dynamic changes between inflammation and immune response, mainly manifested in inflammation induced immunosuppression. Tumor cells secrete IL-10, TGF-β and other immunosuppressive molecules. The uptake of immune factor molecules by surrounding cells such as macrophages, mast cells and natural killer cells stimulate these cells to secrete inflammatory mediators such as IL-10, IL-6 and TNF-α, forming a vicious cycle to strengthen the inflammatory microenvironment (13). Various immunosuppressive cells and inflammatory-related factors are recruited to further secrete tumor-associated inflammatory cytokines and participate in tumor immune escape. Many inflammatory cell types display both tumor-promoting and tumor-suppressive capabilities during tumor development. Further study of the distinctions between the pro-tumor and anti-tumor activities of inflammatory cell is warranted to develop more effective immunotherapies (14).

## The Cytokine Network in the Inflammatory Immunosuppressive Microenvironment of Pancreatic Cancer

Cytokines are produced by inflammatory cells (e.g., B and T lymphocytes, macrophages and mast cells), stromal cells in TME and tumor cells (15). Cytokines include chemokines, interferons, interleukins, lymphokines and TNF. The critical roles of cytokines in the context of inflammation have gained special interest. Dysregulation of the complex interactions between pro- and anti-inflammatory cytokines form a pro-tumorigenic microenvironment with an adverse effect on cancer cell proliferation, invasion and drug resistance (Summary in **Table 1**). Re-establishment of cytokine homeostasis in cancer may provide benefit for patients (86).

**Table 1:** The cytokine network in the inflammatory immunosuppressive microenvironment of pancreatic cancer.

Cytokine	Cell sources	Effects on inflammatory factors	Effects on immune cells	Effects on cancer growth	Refs.
IL-1	APCs; cancer	Inducing IL-6, IL-17 and CXCL8 expression	Fostering an immunosuppressive micromilieu via recruitment of Treg, TAMs and MDSCs	Promoting PC growth by activating PSCs and CAFs	(16–19)
IL-6	Cancer; CAFs; macrophage	Inducing IL-10, IL-7, COX2 and PEG2 synthesis	Maintaining the balance between the regulatory subclass of Treg and Th17	Increasing PC cell migration and invasion via MAPK and PI3K	(20–25)
IL-8	Cancer, macrophages, neutrophils, lymphocytes	Activating STAT/ERK, NF-κB and p38 MAPK signaling	Contributing to tumor immunosuppression formation by recruiting MDSCs and N2 tumor-associated neutrophils (TANs)	Stimulating cancer cells proliferation via interacting with CXCR1 and CXCR2	(26–32)
IL-10	Immune cells; TAMs	Decreasing IL-12 and IFN-γ expression	Inhibiting immune response by interference in DCs and macrophage activation as well as suppressing APCs function	Having both tumor-promoting and tumor- suppressive effects	(33–39)
IL-17	Th17	Inducing secretion of IL-1 β, IL-6, IL-12 and TNF-α.	Producing an immunosuppressive microenvironment by enhancing activity of MDSCs	Playing a dual role in tumorigenesis	(40–51)
INF-γ	CTLs, NK and macrophages	Inducing TNFα and IL-6 production	Increasing tumor immunogenicity by upregulation of MHCI	(1) Inhibiting tumor growth via recruiting CTLs; (2) promote tumor development by enhancing a Th17 reaction	(52–58)
TNF-α	Tumor; inflammatory cells	Increasing IL-1β, IL-6, IL-8, IL-17 and COX2	Impairing immune surveillance by suppressing T cell and the cytotoxic activity of macrophages	promoting tumorigenesis by production of ROS, RNS and MMPs	(59–62)
CXCL10	PSCs, cancer cells, inflammatory cells	Contributing to an inflammatory microenvironment via CXCL10/CXCR3 signaling	Inducing tumor immunosuppression by recruitment of CXCR3^+^ Tregs	Promoting tumor growth, migration and invasion of cancer cells	(63–66)
TGF-β	Tumor; PSCs	Increasing IL-10, SOX4, miR-100 and miR-125b	Promoting immune escape by inhibiting DC maturation and reducing expression of MHC-II and CD80	Enhancing tumor cell progression via inducing EMT	(67–72)
HMGB1	Necrotic cells; immune cells	Inducing secretion of IL-6, IL-8 / CXCL-8, HIF1α, NRP1 and GRO-α/ CXCL-1	Regulating DNA damage repair and inducing Th1 response	Promoting tumor proliferation, angiogenesis, EMT, and metastasis	(73–77)
HIF-1	Tumor cells	Inducing of VEGF, PDGF, TGF-β and ET-1	Contributing tumor immune escape by increasing CTLA-4 expression on CD8+ T cells and PD-L1 expression on cancer cells	Promoting gemcitabine resistance in pancreatic cancer by increasing glycolytic flux and de novo pyrimidine biosynthesis	(78–83)
VEGF	TAMs	Inducing secretion of IL-6, IL-8 / CXCL-8, HIF1α, NRP1 and GRO-α/ CXCL-1	Promoting inflammation and immunosuppression by activating TAMs	Leading to a metabolic transition from mitochondrial oxidative phosphorylation to glycolysis in pancreatic cancer	(84, 85)

IL-1 is a pro-inflammatory cytokine produced by antigen-presenting cells (APCs) and is frequently upregulated in several cancers and chronic inflammatory diseases. IL-1 induces the expression of IL-6 and CXCL8 (16). IL-1 is required for the polarization of IFN-γ-producing CD8^+^ T cells. Furthermore, IL-1 stimulates IL-17 production and generates anti-tumor T cells. Cancer cell–derived IL-1α induces the recruitment of regulatory T (Treg) cells to foster the formation of an immunosuppressive micromilieu by increasing CCL22 expression (17). IL-1 facilitates tumor growth by inducing angiogenesis and the recruitment of MDSCs to the tumor site. Das et al. reported that IL-1β was essential for the establishment of the pro-tumorigenic tumor microenvironment in pancreatic cancer. Tumor cell–derived IL-1β activates PSCs and establishes an immunosuppressive milieu mediated by M2 macrophages, MDSCs and Th17 cells. Neutralization of IL-1β will enhance the anti-tumor activity of PD-1 and be accompanied by increased infiltration of CD8^+^ T cells (18). As tumor-secreted ligands, IL-1 and TGF-β promote CAFs heterogeneity in the TME of pancreatic cancer. IL-1 induces leukemia inhibitory factor (LIF) expression and JAK/STAT activation to generate inflammatory CAFs, and TGF-β antagonizes this process by downregulating IL-1R1 expression and promoting differentiation of CAFs into myofibroblasts (19).

IL-6 is a proinflammatory cytokine produced by various cells including pancreatic cancer cells, hepatocytes and macrophages. IL-6 induces COX-2 expression and PGE2 synthesis in response to infection (20). In the pancreatic TME, IL-6 negatively regulates apoptotic processes *via* MAP/STAT pathway and AKT/PI3K signaling cascade, making cells more resistant to death. IL-6 also maintains the balance between the Tregs and Th17 cells that produce IL-17, IL-6, TNF-α and other pro-inflammatory chemokines (21). IL-6 plays various roles in the progression of pancreatic cancer. Both pancreatic cancer cells and CAFs produce IL-6, which causes increased tumor cell migration and invasion, as well as epithelial to mesenchymal transition (EMT) (22). Although a variety of molecules including mesothelin and advanced glycation end product receptor affect the expression of IL-6, the most important enhancer is KRAS (23). The IL-6 pathway starts JAK kinase (JAK)-1 and JAK-2, resulting in the phosphorylation of STAT-1 and STAT-3. IL-6 signals activate MAP kinase (MAPK) and phosphatidylinositol 3-stimulation (PI3K), which are associated with anti-apoptotic and carcinogenic functions. In addition, IL-6 promotes the formation of a tumorigenic TME by enhancing the expression of IL-10, IL-13, IL-5, IL-7 and granulocyte and macrophage stimulating factors. In patients with pancreatic cancer, a high level of serum IL-6 is associated with cachexia, resulting in promoted niche formation in pancreatic cancer liver metastasis and a poor prognosis (24). The combined treatment of anti-IL-6R with anti-programmed death-ligand 1 (PD-L1) immunotherapy can decrease tumor growth, reduce the abundance of α-smooth muscle actin (α-SMA) ^+^ fibroblasts and increase the infiltration of effector T-cells (25).

IL-8, known as C–X–C motif ligand 8 (CXCL-8), is produced by monocytes, macrophages, neutrophils, lymphocytes, fibroblasts, endothelial cells, and several types of cancer cells (26). IL-8 was first described as a neutrophil chemoattractant in 1989 (27). IL-8 participates in infection response and the pathogenesis of cancers by interacting with specific cell surface G protein–coupled receptors CXCR1 and CXCR2, which leads to the recruitment of neutrophils, stimulation of angiogenesis and stimulation of tumor cell proliferation. Moreover, IL-8 has been shown to be involved in cancer-induced cachexia in pancreatic cancer by activating STAT/ERK, NF-κB and p38 MAPK signaling (28). IL-8 is a pro-inflammatory chemokine, and its expression is stimulated by various cytokines including IL-1, IL-6, CXCL12, TNF-α, hypoxia and reactive oxygen species (ROS). In pancreatic cancer, the hypoxic stressed TME stimulate TAMs to secrete IL-8 mediated by NF-κB, leading to IL-8 to increase CXCR1/2 expressing in endothelial cells and enhance tumor angiogenesis. Endothelial cells initiate the angiogenic process by secreting matrix metalloproteinases (MMPs) to break down the extracellular matrix (ECM) and promote the formation of capillaries once IL-8 stimulation (29). A recent study showed that IL-8 recruited MDSCs and N2 tumor-associated neutrophils (TANs) to tumor foci in a dose-dependent manner and induced granulocytic MDSCs to release DNA to form neutrophil extracellular traps, which contributed to thrombus formation and tumor immunosuppression in cancer (30). Arginase 1 secreted by N2 TANs recruits Treg cells, restrains T cell receptor expression, decreases antigen-specific T cell responses and induces tumor immune evasion. IL-8 also confers tumor immune escape by inhibiting CTL lysis, inducing autophagy and reducing the formation of immunological synapses *via* EMT. The CXCR1/2 blocking agent reparixin abolishes or reverses the above effects to a certain extent (31). Another study showed that blockade of IL-8 decreased mesenchymal and stemness features of tumors, reduced the recruitment of MDSCs to the tumor site, and increased the anti-tumor efficacy of NK cell or antigen-specific T cell–mediated lysis (32). Therefore, IL-8-CXCR1/2 pathways may be an attractive therapeutic strategy for the development of targeted molecular treatment for tumors.

IL-10 is a cytokine with immunosuppressive effects *via* inhibiting APCs activity that is produced by a variety of inflammatory cells. There are many mechanisms to explain the inhibition of APCs function mediated by IL-10, including interference in TLR-mediated or IFN-γ-mediated dendritic cell (DC) and macrophage activation as well as the direct induction of genes that encode proteins to suppress APC function (33). However, the exact mechanism of IL-10 in immunosuppression is not fully understood. IL-10 inhibits the proliferation of antigen-specific CD4^+^ T cells by reducing the expression of MHCII molecules and costimulatory molecules CD80 and CD86 on the surface of APCs such as DCs and macrophages. Mittal et al. found that the expression of IL-10 in APCs was mainly mediated by phosphorylated STAT3, which inhibited the MAPK pathway, PI3K/Akt pathway and NF-κB pathway, interfered with TLR signal transduction and inhibited T cell function (34). IL-10 shows both tumor-promoting and tumor-suppressive effects in the development and pathogenesis of tumors. Three major activities of IL-10 contribute to these paradoxical outcomes: 1) promoting the proliferation and activity of cytotoxic T-lymphocytes (CTLs); 2) inhibiting antigen presentation and production of proinflammatory cytokines from APCs; and 3) alleviating chronic inflammation–mediated tumor promoting effects (35). IL-10 production by TAMs can blunt anti-tumor responses by inhibiting the functions of APCs and subsequently blocking T cell effector functions (36). IL-10 also suppresses tumor-infiltrating DC maturation and their production of IL-12 to stimulate Th1 cells (37). Anti-IL-10R antibody converts tumor infiltrating suppressive macrophages to an active state *via* a DC- and T cell–dependent mechanism (38). High serum IL-10 level has been linked with late-stage cancer and negative prognosis. However, increased expressions of IL-10 are often accompanied by production of other cytokines during inflammation and may not reflect systematical immunosuppression (35). Conversely, IL-10 has been reported to have potent anti-tumor effects *via* inhibiting macrophages and angiogenic factors and activating CD8^+^ T cell. Previous studies reported that IL-10 activated CD4^+^ T cells and CD8^+^ CTLs under certain *in vitro* conditions. Administration of pegylated IL-10 resulted in the rejection of implanted tumors, accompanied by an increase in the number and functions of tumor-infiltrating CTLs (39). Manipulating IL-10 pathways may provide therapeutic benefits for pancreatic cancer patients.

The IL-17 proinflammatory cytokine is produced by the Th17 sub-population of T lymphocytes. After binding to its receptor, IL-17A activates the MAPKs, ERK1/2, p38, PI3K/Akt and NF-κB pathways, leading to the production of TNF-α, IL-1β and IL-6 which attract neutrophils (40, 41). TNF-α and IL-6 not only support Th17 cell development but also synergize with IL-17 to enhance the production of proinflammatory mediators. IL-17 plays a dual role in tumorigenesis. IL-17 induces T cells, macrophages, epithelial cells and endothelial cells to secrete IL-1β, IL-6, IL-12, TNF-α and other inflammatory cytokines to promote the expression of chemokines, such as CXCL-18, CXCL-1 and MCP-1. This results in anti-tumor effects by enhanced activation of NK cells and CTLs and recruitment of neutrophils, NK cells, CD4^+^ and CD8^+^ T cells (42, 43). Moreover, IL-17 also inhibits tumor growth by increasing the generation and activity of CTLs. However, other studies show that IL-17 expression is positively correlated with the invasiveness of tumors. IL-17 induces a wide range of angiogenic mediators, including VEGF, IL-8 and IL-6, to promote tumor growth (44, 45). The IL-17 produced by macrophages has suppressive effects on anti-tumor T cell responses. Monocyte-derived macrophages shift towards a M2-like phenotype upon IL-17A or IL-17F stimulation *via* NF-κB activation (46, 47). Wu et al. reported that the IL-17B-induced IL-17RB pathway activated downstream cytokine gene targets that promoted oncogenesis and metastasis of pancreatic cancer *via* NF-κB and MAPK pathways (48). In tumor-bearing mice, IL-17 promotes the formation of an immunosuppressive microenvironment by reversing the gene expression inhibition normally maintained by the mRNA decay factor AUF1, thus promoting tumor development and enhancing the activity of MDSCs (49, 50). In patients with various types of gastrointestinal cancer, IL-17 production is correlated with MDSC levels and identified as a sensitive marker for nutritional impairment, immune suppression and chronic inflammation (51).

IFN-γ is a multifunctional cytokine family with broad-spectrum antiviral, antiproliferative and immunomodulatory activities. It is produced mainly by CD8^+^ CTLs, natural killer cells and macrophages. INF-γ induces TNF-α and the production of cytokines such as IL-6 which mediates the Th1 inflammatory response. The IFN-γ inflammatory cytokine has a pivotal role in anti-infection and tumor immune surveillance. IFN-γ promotes cellular immunity against infections and inhibits the growth of cancer cells. IFN-γ is involved in anti-proliferative, antiangiogenic and pro-apoptotic effects against cancer cells. It increases tumor immunogenicity by upregulation of MHC I genes. Recognition and elimination of tumor cells by CTLs are recruited to the tumor mass *via* IFN-γ–induced chemokine signaling (52). A clinical trial showed that IFN-γ in the first-line treatment of ovarian cancer improved the progression-free survival of patients (53). However, some evidence has indicated that the IFN-γ cytokine exhibits both anti- and pro-tumorigenic functions (54). IFN-γ promotes tumor development by enhancing a Th17-associated inflammatory reaction (55). Zhang et al. reported that IFN-γ induced the expression of PD-L1 by the PI3K/AKT and JAK/STAT3 signaling pathways, thereby promoting the immune escape of tumor cells (56). A recent study showed that IFN-γ was involved in tumor promotion by upregulating the number of immunosuppressive cells *via* inducing the expression of indoleamine 2,3-dioxygenase (IDO), thereby increasing the number of Tregs and decreasing the activity of CTLs (57, 58). The application of IFN-γ as a therapeutic agent for cancer treatment should be explored with caution, considering that role of INF-γ in malignant tumors is complicated.

TNF-α is produced by tumor and inflammatory cells within the TME. TNF-α is the cytokine that most consistently associated with tumor cell killing through activation of the transcription factor NF-κB and subsequent production of IL-1β, IL-6, IL-8 and IL-17. TNF-α and NF-κB interact to induce cytokines (e.g., IL-1, IL-6), COX-2 and MMPs. TNF-α binding to its receptor TNFR upregulates pro-inflammatory cytokines in response to wounding or infection (59). However, TNF-α promotes tumorigenesis and enhances tumor progression in chronic infection by the production of ROS and reactive nitrogen species, deregulation of apoptotic pathways and induction of matrix metalloproteinases (MMPs) (60). TNF-α promotes tumor cell survival through the induction of genes encoding NF-κB-dependent antiapoptotic molecules. TNF-α also contributes to tumor initiation by stimulating the production of genotoxic molecules such as nitric oxide (NO) and ROS. Other roles of TNF-α include the recruitment of Tregs at the cancer site and impairment of immune surveillance by suppressing T cell responses and the cytotoxic activity of activated macrophages (61). Anti-TNF-α antibodies might be an effective anti-cancer therapy. However, reports on the effects of anti-TNF-α treatment have yielded varying results. Two clinical trials in the USA evaluated the effects of TNF-α and TNF-α inhibitors in patients with pancreatic cancer. The results showed that TNF-α and anti-TNF-α therapy provided no clinical benefit in terms of the duration of survival of patients with unresectable pancreatic cancer (3).

CXCL10, also called interferon-γ inducible protein 10 (IP-10), is a chemokine expressed in many inflammatory diseases including pancreatitis. CXCL10 regulates the chemotaxis of CXCR3^+^ immune cells such as macrophages, T cells and natural killer (NK) cells *via* targeting the cognate receptor CXCR3. CXCL10 promotes tumor growth, migration and invasion. Expression of both CXCL10 and CXCR3 in tumor tissue has been correlated with a poor prognosis in pancreatic cancer (62). There is a close association between the expression of CXCL10 and CXCR3 with the presence of Tregs and an immunosuppressed microenvironment. CXCL10 is also positively correlated with M1 macrophages, which act as antitumoral immune components (63). Pandey et al. showed that INF-γ increased the expression of CXCL10 *via* JAK-STAT1 signaling. As a chemoattractant, CXCL10 recruits inflammatory macrophages into pancreatic lesions, which enhance cancer cell proliferation and maintain their inflammatory identity. Blocking CXCL10/CXCR3 signaling results in a loss in M1 polarized macrophages, shifting macrophage populations to a tumor-promoting phenotype (64). Lunardi *et al.* found that pancreatic cancer cells stimulated PSCs to produce CXCL10 which increased the recruitment of CXCR3^+^ Tregs that were involved in inducing tumor immunosuppression (65). Therefore, targeting the CXCL10/CXCR3 signal axis could not stimulate an immune response against tumors because blockade of CXCL10/CXCR3 may decrease T effector cell recruitment and lead to a macrophage polarization shift. Thus, even though CXCL10 has an important effect on pancreatic cancer, new multimodal therapeutic approaches need to be explored.

TGF-β is a secretory multipotent factor that plays an important role in regulating cell proliferation, differentiation and migration. TGF-β signals have a dual action in cancer, and its role in tumor suppression and tumor progression depends on the cellular context. During pre-malignant states, TGF-β exerts tumor-suppressive effects on tumor cells. Once tumor cells circumvent the suppressive effects of TGF-β, they use TGF-β to their advantage to enhance tumor cell progression (66). David et al. showed that TGF-β promoted tumor suppression in pancreatic cancer cells by promoting EMT-linked transcription factor landscape, which converted SOX4 from an enforcer of tumorigenesis in the epithelial state into a promoter of apoptosis after EMT (67). TGF-β and RAS signaling synergize to induce SNAIL expression, which is coupled to apoptosis owing to an imbalance of SOX4 and KLF5 transcription factors. Inhibitor of differentiation 1 (ID1) uncouples TGF-β-induced EMT (68). Other studies indicate that TGF-β promotes tumorigenesis and metastasis at the advanced stage of pancreatic cancer by inducing miR-100 and miR-125b (69). TGF-β is also an important factor that induces M2 polarization of macrophages. M2 macrophages are common in the TME and closely related to TGF-β. TGF-β promotes the secretion of IL-10, reduces the expression of DC surface molecules such as MHC-II molecules and CD80, inhibits DC differentiation and maturation and makes it unable to activate T cells. By activating Smad, TGF-β inhibits the production of cytokines such as perforation protein and caspase activated secretory factors granzyme A and B and promotes apoptosis factors FasL and FasL to induce immune escape. TGF-β also inhibits the proliferation of T cells and B cells. Mariathasan et al. found that TGF-β was highly expressed in tumor tissues of patients who showed no response to the immune checkpoint inhibitor atezolizumab, suggesting that primary drug resistance against the immune checkpoint inhibitor might be related to the TGF-β pathway (70). Therefore, TGF-β inhibitors combined with PD-L1 inhibitors can reshape the matrix microenvironment and induce T cells to infiltrate tumors. By co-inhibition of the TGF-β pathway and the PD-L1 checkpoint, tumor growth is significantly inhibited with enhanced CD8^+^ T cell infiltration and increased production of IFN-γ (71). However, no clinical evidence is available to support the combination of TGF-β inhibitors with PD-L1 inhibitors over the single drug treatments. The specific efficacy and mechanism require further study.

High mobility group box 1 (HMGB1) is released from necrotic cells or secreted by inflammatory cells into the local microenvironment. Extracellular HMGB1 promotes inflammation by stimulating neutrophils or monocytes to produce inflammatory cytokines and chemokines (72). Moreover, HMGB1 activates endothelial cells, enhances angiogenesis, and induces migration of immune and stem cells, thereby initiating an inflammatory response (73). HMGB1 secreted by dying tumor cells triggers the activation of IFN-γ polarizing tumor-antigen specific T-cells through a TLR4- and MyD88-dependent mechanism (74). HMGB1 plays dual roles in the development of cancer. HMGB1 can contribute to tumorigenesis, as HMGB1 produced by tumor cells may exacerbate inflammation-related immunosuppression. In addition, HMGB1 promotes the release of IL-6 and IL-8 by activating MAPK- and MyD88-dependent NF-κB pathways, resulting in promoted tumor proliferation, angiogenesis, EMT, invasion and metastasis (75). HMGB1 also plays a protective role in tumor suppression and immunotherapy by regulating DNA damage repair and inducing the Th1 response (76). HMGB1 has complex roles in cancer progression, depending on its subcellular localization, post-transcriptional modification and binding receptors.

Hypoxia inducible factor-1 (HIF-1) is activated and induced in the hypoxic TME, and coordinates a transcriptional program that ensures metabolic adaptation to O_2_ shortages. Tumor cells promote HIF-1α synthesis by activating PI3K and MAPK signaling pathways. The target genes regulated by HIF-1 include the vascular endothelial growth factor (VEGF), endothelin-1 (ET-1), insulin-like growth factor II, and platelet-derived growth factor (PDGF) genes (77, 78). HIF-1α induces vascular target genes, and VEGF is the main target to induce tumor angiogenesis and improve blood flow in inflammatory reaction sites or tumor tissues. HIF-1α also promotes pancreatic cancer cells to secrete TGF-β, a critical modulator of inflammation and an important chemoattractant for Treg recruitment into tumors. HIF-1α decreases the susceptibility of cancer cells to T-cell-mediated cytotoxicity, contributing to tumor immune escape by increasing CTLA-4 expression on CD8^+^ T cells and PD-L1 expression on hypoxic cancer cells (79). Hypoxia and HIF-1 mediate activation of autophagy in tumor cells and regulate natural killer (NK) cell–mediated antitumor responses. HIF-1 also induces the expression of genes encoding glycolytic enzymes and glucose transporters such as GLUT1 and hexokinase (HK), thus enhancing glycolytic intermediates such as pyruvate and lactate by the action of lactate dehydrogenase and pyruvate dehydrogenase kinase (PDK) to be made up (80). Shukla et al. reported that HIF-1α mediated increased glycolytic flux and *de novo* pyrimidine biosynthesis, leading to gemcitabine resistance in pancreatic cancer cells. Targeting HIF-1α biosynthesis increases the efficacy of gemcitabine (81). HIF inhibitors may exert cytotoxic effects or reduce the resistance to treatment (chemotherapy, radiotherapy and immunotherapy), which are emerging as a potential therapeutic modality in cancer (82).

VEGF is a key factor related to tumor promoting inflammation and angiogenesis and has immunosuppressive functions. VEGF promotes the release of inflammatory cytokines through VEGFR2, which induces the secretion of IL-6, IL-8/CXCL-8 and GRO-α/CXCL-1 in endothelial cells. Inflammatory cytokines such as TNF-α, IL-1B, IL-6 and IL-8/CXCL-8 induce the expression of VEGF (83). VEGF promotes inflammation and immunosuppression by activating TAMs, which are an important source of VEGF. Therefore, a positive feedback loop is established between inflammation/angiogenesis and immunosuppression. As an important target gene of HIF1α, VEGF also plays important roles in the regulation of glucose metabolism in pancreatic cancer. VEGF promotes the metabolic transition from mitochondrial oxidative phosphorylation to glycolysis in pancreatic cancer. VEGF treatment increases the expression of HIF1α and neuropilin 1 (NRP1) as well as those of glycolytic enzymes. NRP1, a co-receptor for VEGF, plays a key role in VEGF-induced glycolysis through HIF1α upregulation (84). Drugs targeting VEGFR2 or NRP1 may inhibit pancreatic cancer glycolysis and represent a new strategy to treat pancreatic cancer.

## Intercross Between Immunosuppression and Inflammatory Reaction in the Pancreatic TME

**Figure 1:**
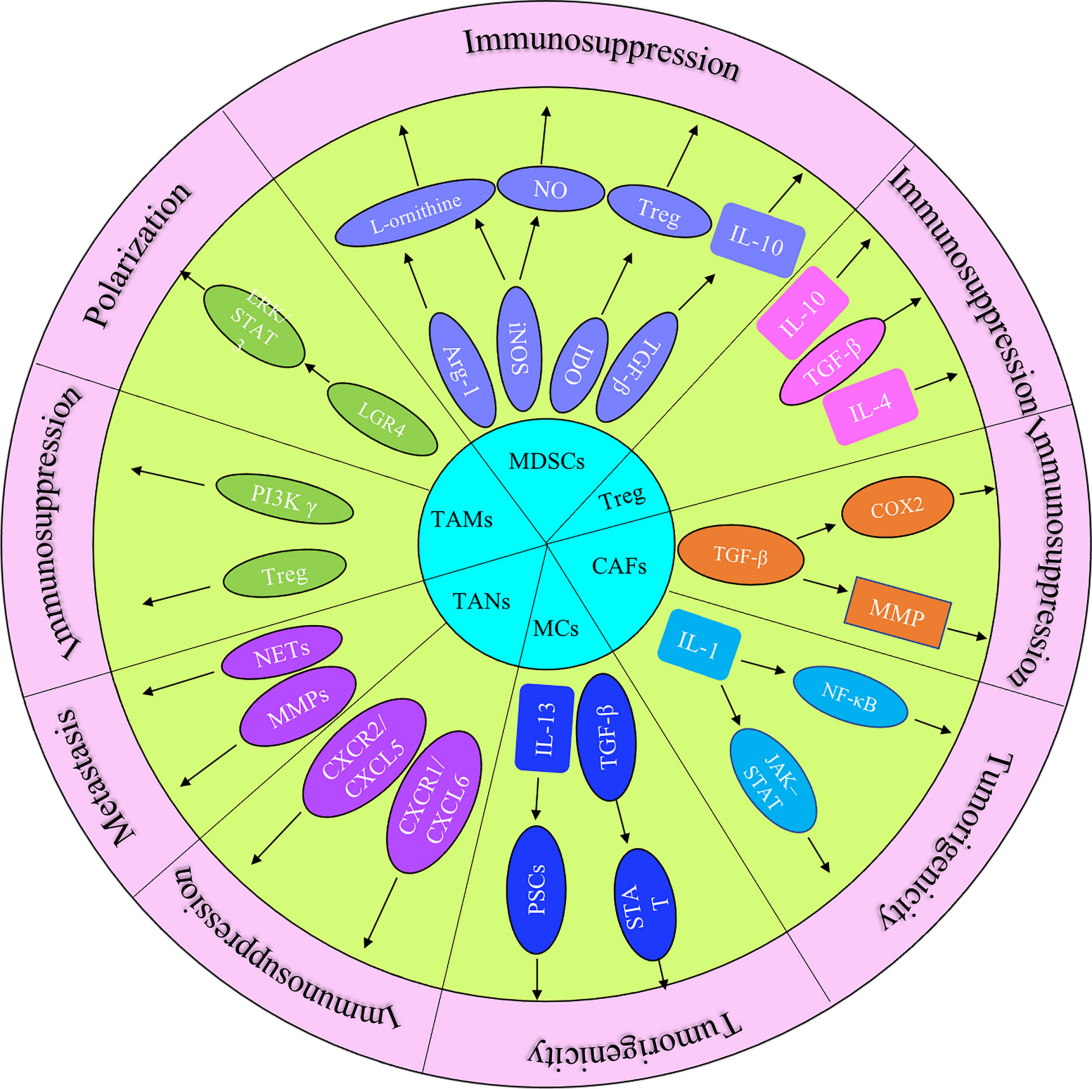
The role of principal cytokines and stromal cells (MDSCs, CAFs, TAMs, TANs, Treg and MCs) that coordinate the tumor-promoting and immunosuppressive effects in pancreatic cancer progression.

**Figure 2:**
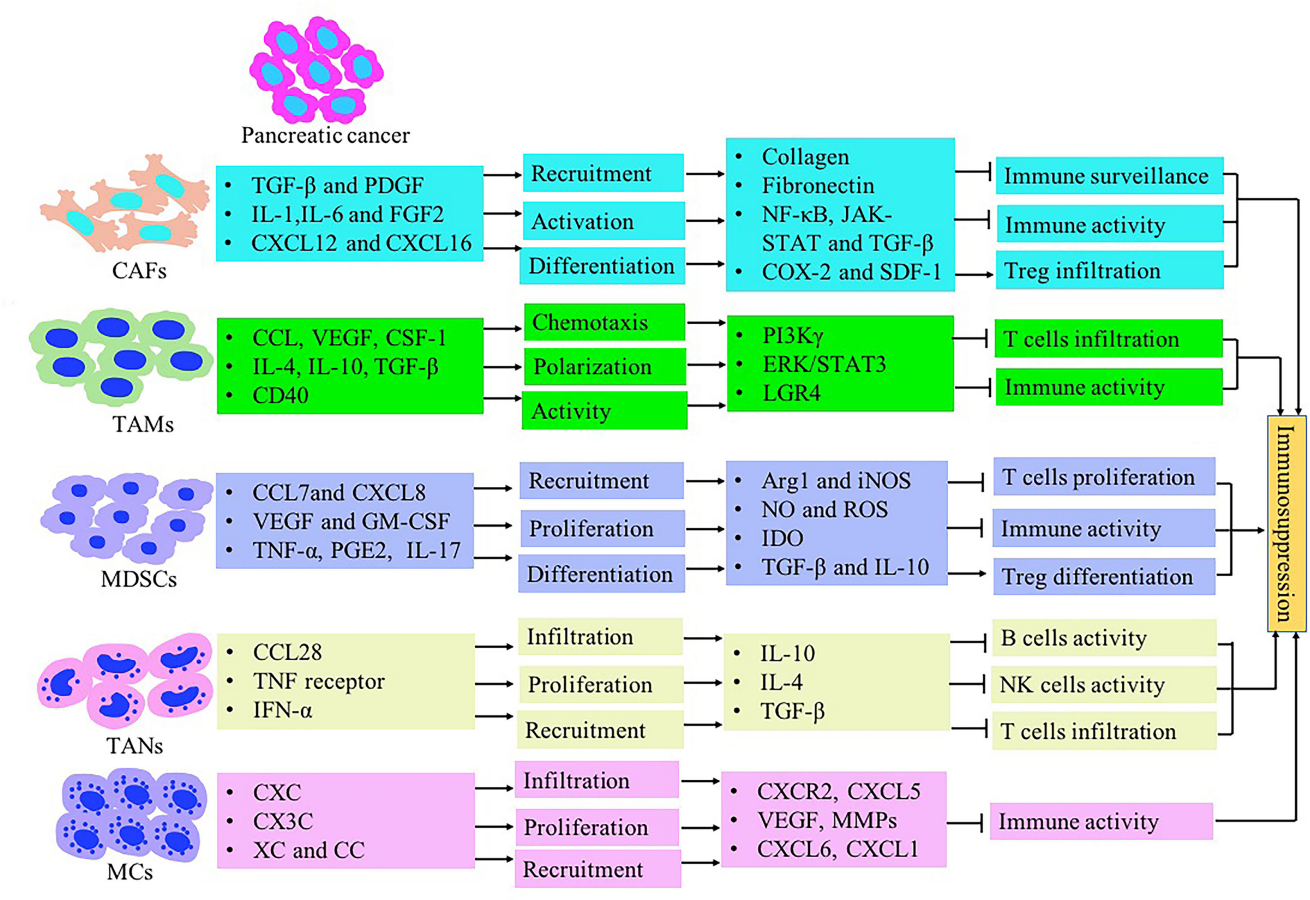
The immunosuppressive tumor microenvironment in pancreatic cancer mediated by stromal cells such as CAFs, MDSCs, TAMs, TANs, Treg and MCs. Inflammatory-associated factors secreted by pancreatic cancer induce stromal cells recruitment and expansion in the pancreatic TME. Stromal cells contribute to immunosuppression formation through inhibiting T lymphocyte activity and inducing Treg cells.

The immune response in the TME is regulated by the balance between effector T cells and Tregs. An imbalance of the immune response in the TME leads to the inhibition of the recruitment of immune cells to tumor site. It may also promote tumor growth through the secreted cytokines (**Figure 1**). The microenvironment of pancreatic cancer is infiltrated with different types of immune cells. They are in an imbalance state of quantity and function under the induction of tumor cells. The presence of large numbers of immunosuppressive cells creates an immunosuppressive microenvironment, which is beneficial for pancreatic cancer cells to evade immune surveillance, thereby contributing to the proliferation, invasion and metastasis (**Figure 2**).

## Cancer-Associated Fibroblasts (CAFs)

CAFs are the dominant cells of pancreatic TME and used as immunosuppression biomarkers. CAFs are different from normal fibroblasts and express specific markers such as fibroblast activating protein (FAP), α-SMA, fibroblast specific protein and platelet-derived growth factor receptor. The recruitment, activation and reprogramming of CAFs are induced by a variety of signals, including growth factors (TGF-β, PDGF and FGF2), cytokines (IL-1 and IL-6/IL-11), chemokines (CXCL12 and CXCL16), hypoxia and ECM components (85). TGF-β and IL-1 are two well studied important cancer cell–derived molecules. TGF-β plays a crucial role in the differentiation of CAFs into a myofibroblast-like phenotype. The communication between CAFs and TME is meditated by TGF-β, and CAFs can release extracellular matrix components such as collagen and fibronectin to activate TGF-β. Mechanisms of CAF-mediated immunosuppression by TGF-β stimulation include immune cell exclusion, inhibition of the proliferation of anti-tumor cells and resistance to immunotherapy (70). IL-1β induces an NF-κB-mediated proinflammatory signature in CAFs. IL-1α activates inflammatory CAFs in pancreatic cancer through the JAK-STAT pathway and is antagonized by TGF-β (19). CAFs also have immunosuppressive properties by upregulating the expression of histone deacetylase through a COX-2- dependent mechanism. Activated CAFs secrete chemokines such as SDF-1 to recruit CD8 ^+^ T cells, thus blocking their access to tumor cells (87). When more CD8^+^ T cells are recruited into the tumor matrix, eliminating FAP^+^ CAFs reactivates the antitumor immune response. Other studies showed that α-SMA^+^ CAFs with stronger tumor invasiveness increased infiltration of Tregs and inhibited immune surveillance. The development of a treatment targeting CAFs has received attention, but it still faces some challenges, including the following: 1) the source of CAFs is uncertain; 2) targeted CAFs are heterogeneous in phenotype, secretory spectrum and subgroup function, which brings difficulties in diagnosis and targeted therapy; 3) CAFs induced signals have functional diversity; CAFs can activate signals that play a role both in tumorigenicity and anti-tumorigenicity; and 4) the function of CAFs varies with tumor type (88). In depth genome sequencing can analyze the molecular structure of CAFs and accelerate the development of CAFs specific diagnosis, prognosis evaluation and CAFs targeted therapy. Therefore, it is necessary to conduct more in-depth research on the different subtypes of CAFs, determine the dominant subtypes in tumor progression and focus on their targeted treatment.

## Tumor-Associated Macrophages (TAMs)

TAMs with strong plasticity are the most common infiltrated immune cells in the pancreatic TME. TAMs mainly come from monocytes in peripheral blood. Pancreatic cancer cells and stromal cells secrete large numbers of chemokines to induce monocytes in blood circulation to enter tumor tissue. Chemokines that recruit monocytes into tumor tissues include CCL family proteins, VEGF, colony stimulating factor (CSF)-1, placental growth factor and monocyte chemotactic protein-1 (MCP-1, also known as CCL2). Activated macrophages are classified into two phenotypes: 1) classically activated macrophages, also known as M1 macrophages, which are induced by bacteria and their products lipopolysaccharide and interferon-1; these cells show high antigen presentation ability, secrete IL-12, NO and IL-10, and are involved in the Th1 immune response and killing tumor cells; and 2) alternative activated macrophages, known as M2 macrophages, which are activated by IL-4, IL-10 and TGF-β (89). These macrophages have strong plasticity, and cytokines in TME affect differentiation of macrophages. TAMs can achieve M1/M2 type transformation under different stimulating factors; this provides a theoretical basis for the targeted remodeling of TAM, “educating” the transformation of macrophages from the M2 type to the M1 type with the purpose of inhibiting pancreatic tumor growth and metastasis (90). Macrophage colony stimulating factor 1 (CSF-1) plays an important regulatory role in the differentiation, polarization and chemotaxis of macrophages. CSF1/CSF1R signaling regulates the number and the function of TAMs, and their activities depend on tumor-type/tissue-specific factors (91). CD40 activates macrophages to infiltrate rapidly around the tumor cells, weaken the immunosuppressive effect in the TME and drive T cells to produce anti-tumor responses (92).

TAMs in the pancreatic TME lose the functions of presenting tumor-related antigens, killing tumor cells and activating T lymphocytes. TAMs induce Treg lymphocytes, mediate inhibition of antitumor activity, increase tumor growth and reduce survival rate. TAMs exert an immunosuppressive role *via* the PI3Kγ signal pathway. Macrophages activate the PI3Kγ signal pathway, inhibit T cell activation, and block PI3Kγ signal pathway can enhance the effect of anticancer drugs and immunotherapy (93). In addition, LGR4, a member of the G-protein coupled receptor (GPCR) family, plays a key role in the regulation of tumor immunity. LGR4 promotes the differentiation of M2 macrophages by activating the ERK/STAT3 signaling pathway. Knockout of LGR4 significantly inhibits the tumor growth and prolongs the survival time, and increased numbers of M1 macrophages and activated CD8 ^+^ T cell infiltration are also observed, suggesting that LGR4 promotes antitumor effects by changing macrophage polarization (94). Gemcitabine combined with a chemokine receptor CCR2 or CSF-1 receptor inhibitor to reduce the number of TAMs in pancreatic TME increases the number of CD8^+^T lymphocytes, reduces FOXP3^+^ Treg lymphocytes and inhibits the development of pancreatic cancer, indicating that reducing TAMs increases the responsiveness of pancreatic cancer to anti-tumor immunity (95, 96).

## Myeloid-Derived Suppressor Cells (MDSCs)

MDSCs play an important role in the progression of pancreatic cancer. MDSCs are a heterogeneous cell population derived from bone marrow progenitor cells and immature bone marrow cells (IMCS). Under the pathological conditions of tumors, various infectious diseases and autoimmune diseases, IMCS cannot differentiate into mature bone marrow cells but instead produce MDSCs with immunosuppressive function (97). The recruitment of MDSCs is a complex process and regulated by a variety of chemokines. CCL2 and CCL5 secreted by tumor cells are the two main chemokines. Studies have shown that CCL7, CXCL8 and CXCL12 also recruit m-MDSCs to tumor sites (98). The activation of MDSCs depends on the participation of cytokines. Studies have shown that growth factors play an important role in inducing the aggregation and activity of MDSCs. VEGF, which often exists in the tumor microenvironment and is upregulated in hypoxia, promotes tumor growth by promoting angiogenesis. VEGF is a chemical attractant for MDSCs. Some studies have found that MDSCs in mice produce VEGF, indicating that MDSCs can induce and activate more MDSCs by secreting VEGF (99). Granulocyte macrophage colony stimulating factor (GM-CSF) and granulocyte colony stimulating factor (G-CSF) also promote the aggregation and immunosuppressive function of MDSCs (100). In addition to growth factors, there are also large numbers of inflammatory factors in the TME. TNF-α, prostaglandin E2 (PGE2), IL-6 and IL-1β are important inflammatory factors that increase the number of MDSCs and promote their inhibitory activity (101). Through experiments using PGE2 receptor inhibitors to block the production of PGE2, PGE2 receptor knockout mice or nonsteroidal anti-inflammatory drugs, researchers found that PGE2 promoted the differentiation of mouse bone marrow progenitor cells into MDSCs, and the induction of m-MDSCs occurs through the p38 MAPK/ERK pathway. PGE2 also enhances its immunosuppressive activity by increasing the content of arginase 1 (ARG1) in MDSCs. Other studies have shown that IL-17 increases the numbers of MDSCs in the TME and increases intracellular ARG1, cyclooxygenase 2 and the immunosuppressive molecule IDO1, thus promoting MDSC activation (51).

After being recruited into the TME and activated, MDSCs mediate immunosuppression in the TME through two main mechanisms. One is the consumption of nutrients in the TME; MDSCs consume essential amino acids in the extracellular matrix by expressing Arg1, IDO1 and inducible nitric oxide synthase (iNOS) to hinder the proliferation of immune cells. MDSCs also highly express Arg1 and iNOS, and both enzymes use L-arginine as substrate. Arg1 converts arginine to urea and L-ornithine, and iNOS converts arginine to a large amount of NO and L-citrulline. The results of these two enzyme-catalyzed reactions consume L-arginine in the TME, and L-arginine deficiency hinders the proliferation of T cells and downregulates CD3, a component of T cell receptor (TCR) ζ (102). Another enzyme highly expressed induced by MDSCs is IDO, the key enzyme in the tryptophan degradation pathway. Overexpression of IDO results in decreased tryptophan in the TME and the accumulation of toxic metabolite guanosine, which has an adverse impact on immunity. The activation of IDO also promotes the differentiation of T cells into Tregs. IDO enhances the immunosuppressive activity of Tregs and protects them from reprogramming into helper T cells. The second mechanism is to cause oxidative stress in the TME, producing NO and ROS through iNOS and NADPH oxidase (NOX2), thus affecting the activity of immune cells. MDSCs regulate the T cell immune response by producing oxidative active substances such as iNOS, Arg1 and NOX2 (103). NO inhibits the expression of MHC II and tumor immunity. ROS and reactive nitrogen produced by MDSCs results in the nitrosation of TCR tyrosine sites, thus affecting the activity of CD8^+^ T cells. As mentioned above, PGE2 stimulates the expansion and differentiation of MDSCs. In addition to the above mechanisms, MDSCs also express or secrete a variety of molecules to cause immunosuppression. MDSCs secrete TGF-β and IL-10, inducing the increase of Tregs, inhibiting the function of NK cells and indirectly inducing immunosuppression. MDSCs also secrete pro-inflammatory proteins such as S100A8 and S100A9, stimulating the recruitment of MDSCs to tumor sites and enhancing the inhibitory activity of MDSCs (104).

In view of the significant inhibitory effect of MDSCs on tumor immunity, developing targeted strategies for MDSCs represents a potential therapeutic strategy for pancreatic cancer treatment. Promoting the differentiation of MDSCs into mature bone marrow cells is one of the most promising methods. All trans retinoic acid, a metabolite of vitamin A, promotes the differentiation of bone marrow cells into DCs and macrophages. The number of MDSCs in bone marrow and spleen of mice treated with all trans retinoic acid receptor antagonist increased, while the number of MDSCs in tumor patients and tumor-bearing mice decreased significantly after treatment with all trans retinoic acid (105). Recent *in vitro* experiments showed that blocking GM-CSF and gemcitabine combined with chemotherapy inhibited the differentiation of MDSCs in pancreatic cancer, thereby improving the function of killer T lymphocytes in pancreatic cancer (96). Therefore, the effect of targeting immune cells alone may be limited, and this strategy may need to be combined with traditional chemotherapy to achieve better antitumor effects.

## Regulatory T Cells (Tregs)

Tregs release cytokines such as IL-10, IL-4 and TGF-β. Tregs also inhibit the function of B cells, NK cells and other immune cells. Under hypoxia, tumor cells recruit Tregs by upregulating the expression of chemokine ligand 28 (CCL28), enhancing tumor immune tolerance and promoting angiogenesis (106). Tregs are abundant in the pancreatic TME. These cells inhibit the recruitment and activation of tumor-associated antigen-specific CD8^+^ T cells. In addition, the infiltration of Tregs in tumor tissues is associated with the degree of pancreatic cancer differentiation and an indicator of poor prognosis. Clinical studies showed that after treatment with rF-CEA (6D) -TRICOM carrier–loaded dendritic cells, the pancreatic cancer patients were treated with anti-tumor vaccine after flow injection analysis. The flow cytometry results showed that the numbers of Tregs in peripheral blood decreased and the number of T lymphocytes increased (107). Other studies showed that competitive anti-glucocorticoid-induced TNF receptor monoclonal antibody combined with IFN-α significantly inhibited the infiltration of Tregs in pancreatic tumor and upregulated the number of CD4^+^ and CD8^+^ T cells. The mechanism of gemcitabine *in vivo* involves reducing the Treg cell activity in tumor-bearing mice and enhancing the anti-tumor activity of mice, resulting in prolonged survival (108). Neoadjuvant therapy reduces the infiltration level of myeloid cells in the tumor and exhibits effective anti-tumor effects. Together these findings suggest that remodeling of Treg immunosuppressive cells combined with traditional chemotherapy can improve the immunity of pancreatic cancer patients. Increasing the uptake of chemotherapeutic drugs into tumor tissues will improve the efficacy of chemotherapeutic drugs.

## Tumor-Associated Neutrophils (TANs)

TANs exhibit both antitumoral and protumor functions. The interaction between TANs and pancreatic cancer cells is complex. Tumor cells and stromal cells secrete large numbers of chemokines, induce monocytes in blood circulation to enter tumor tissue and further induce differentiation into neutrophils. TANs release neutrophil extracellular traps (NET), which activate tumor-associated fibroblasts to promote pancreatic cancer metastasis (109). CXCR2^+^ TANs promote tumor-related neutrophil recruitment to the interstitial microenvironment of pancreatic cancer and induce chemotherapy resistance. In addition, TANs inhibit the activity of immune T cells in pancreatic cancer through the CXCR2/CXCL5 signal axis, thus forming an immunosuppressive microenvironment and promoting cancer progression. The release of azurocidin from TANs can reprogram PSCs, further alter the interstitial microstructures of pancreatic cancer and promote cancer cell proliferation (110). Activated TANs synthesize and release a large amount of VEGF, MMPs, CXCL6 and CXCL1 to promote tumor angiogenesis (111). TANs release neutrophil elastase which promotes tumor cell metastasis by degrading the extracellular matrix and enhancing tumor invasiveness. However, some studies have found that neutrophils secrete large numbers of pro-inflammatory factors, including TNF-α, MIP-α, H_2_O_2_ and NO, which directly kill tumor cells (112). TANs mediate cancer cell cytotoxicity by releasing ROS and neutrophil elastase, and potentiate antitumoral T cell responses by inhibiting TGF-β signaling. After removing neutrophils, the activity of CD8 ^+^ T cells is inhibited and tumor growth is accelerated (113). In the early stage of tumor development, TANs upregulate the expression of costimulatory molecules OX-40L and 4-1BBL to enhance the T cell immune response and increase the cytotoxicity of CD4^+^ T cells and CD8^+^ T cells, to exhibit “tumor inhibition” functions (114, 115). The effect of TANs on the progression of pancreatic cancer is controversial. Its role in the immunosuppressive microenvironment of pancreatic cancer and its specific molecular mechanisms need to be further studied.

## Mast Cells (MCs)

MCs are widely distributed around microvessels under the skin and visceral mucosa, secrete a variety of cytokines and participate in immune regulation. MCs are well known for their primary role in allergic reaction, but it has been ignored as a member of tumor microenvironment. MCs act as effectors of tumor-promoting behavior by releasing pre-tumorigenic molecules and secreting a variety of signal molecules, including epithelial growth factor. MCs also play an important role in the occurrence of tumors and prolong the survival of tumor-bearing immunocompetent hosts (116). Tumor cells facilitate the migration of MCs. Pancreatic cancer cells and PSCs also stimulate the activation of MCs (117). IL-13 and tryptase produced by activated MCs further enhance the proliferation of PSCs and promote the deposition of extracellular matrix through the TGF-β2 pathway in a STAT6-independent manner. Blocking MC migration and function *in vivo* suppressed pancreatic cancer growth and improved patient survival (118). Better understanding of the interaction of MCs and PSCs may lead to the development of new therapeutic methods to inhibit pancreatic cancer progression.

## Clinical Approaches Based on Targeting TME in Pancreatic Cancer

Cytokines in TME are one of the most important effector and messenger molecules in mediating the interaction between inflammation and stromal components in pancreatic cancer. Therefore, manipulating cytokine pathways is an effective strategy to treat cancer progression. The important functions of cytokines in pancreatic cancer are supported by data from some clinical trials. Bruton tyrosine kinase (BTK) is a non-receptor enzyme in the Tec kinase family that is expressed in mast cells and myeloid cells in peri-tumoral inflammatory stroma. BTK-dependent signaling is essential to the maintenance of the TME. BTK inhibition converts M2-like macrophages to an M1-like phenotype, promoting CD8-mediated T-cell cytotoxicity. Overman et al. performed a randomized phase II clinical trial to investigate the effect of BTK inhibition in patients with advanced pancreatic cancer using acalabrutinib, an inhibitor of BTK, alone and in combination with the anti-PD-1 antibody pembrolizumab. The results showed the safety of acalabrutinib as a monotherapy and in combination with pembrolizumab and reductions in granulocytic (CD15^+^) MDSCs; however, the overall response rate and disease control rate were 0% and 14.3% with monotherapy and 7.9% and 21.1% with the combination therapy, respectively (119). A phase IIa study was initiated to assess the safety, efficacy and immunobiological effects of the CXCR4 antagonist BL-8040 (motixafortide) with pembrolizumab and chemotherapy in metastatic pancreatic cancer. BL-8040 increased CD8^+^ effector T cell tumor infiltration, decreased MDSCs and further decreased circulating Tregs. Data suggested that BL-8040 and pembrolizumab improved the benefit of chemotherapy in pancreatic cancer. Patients obtained an objective response rate, disease control rate and median duration of response of 32%, 77% and 7.8 months, respectively, after receiving BL-8040 and pembrolizumab with chemotherapy (120). It is reported that combing mogamulizumab (Treg-depleting anti-CCR4 antibody) and nivolumab (anti-programmed death-1 (PD-1) antibody) showed antitumor activity with an acceptable safety rate, suggesting that targeting both PD-1 and Treg depletion may be a potentially effective option in cancer immunotherapy (121). Single-agent inhibitors of Treg do not have activity in pancreatic cancer. In order to study how the TME is altered by immunotherapy, Lutz et al. compared an irradiated, GM-CSF-secreting, allogeneic pancreatic cancer vaccine (GVAX) given as a single agent or in combination with low-dose cyclophosphamide to deplete Treg in the patients of pancreatic cancer. Results showed that cyclophosphamide given with vaccination altered the balance between the infiltrating effector T cells and Tregs in favor of an effector T-cell response (122). Arshad et al. reported that decreasing pro-angiogenic and pro-inflammatory factors was associated with improved prognosis in patients with advanced pancreatic cancer treated with gemcitabine and intravenous omega-3 fish oil by reducing concentrations of CAFs (123). IFN-α has been used either in monotherapy or in combined modality treatment in cancer. A phase II type trial suggested that the combination IFN-α with cisplatin and 5-FU improves survival in patients with pancreatic cancer compared with a Gastrointestinal Tumor Study Group–type protocol (124). Some clinical trials also showed that adjuvant interferon-based chemoradiation can be safe and tolerable for patients with pancreatic cancer and achieved an improvement in overall survival (125, 126). However, data from an EORTC gastrointestinal tract cancer group trial showed that 5-fluorouracil (5-FU) plus cisplatin with or without α-interferon 2b had little activity with considerable toxicity (127). Sparano et al. provided evidence that IFN-α did not enhance the efficacy of 5-FU in patients with advanced pancreatic cancer (128). As one of the most potent antigen-presenting cells of the immune system, DCs are being investigated in clinical trials for their role in stimulating the immune system. A phase I/II clinical trial using DCs transfected with cDNA of the human tumor antigen mucin (MUC1) showed that immunologic responses against tumors were induced in patients, even though the immune responses were not sufficient (129). Mayanagi et al. performed a phase I pilot study to assess the feasibility of immune response to Wilms tumor gene 1 (WT1) peptide-pulsed DC vaccination combined with gemcitabine. The results demonstrated that WT1 peptide-pulsed gemcitabine is feasible and effective for inducing anti-tumor T-cell responses in patients with advanced pancreatic cancer (130). A phase I/II trial was performed to assess safety and efficacy of the toll-like receptor 2/6 agonist MALP-2 in combination with gemcitabine in patients with pancreas carcinomas. Results showed that MALP-2 improved the mean survival significantly by activating monocytes, macrophages and anergic DC (131). Lin et al. reported that allogeneic NK cells combined with irreversible electroporation for advanced pancreatic cancer produced a synergistic effect, not only exhibiting good short-term outcome and improving the quality of life, but also increasing the median progression-free survival (PFS) and median overall survival (OS) (132, 133). Despite these encouraging results, some concerns regarding drug-associated toxicities remain. Approaches to minimize toxicity while preserving efficacy are required.

## Conclusions and Perspectives

Pancreatic cancer cells, stromal cells and cytokines interact to form an inflammatory and immunosuppressive tumor microenvironment. Increasing studies have highlighted the significance of the crosstalk between inflammation and stromal components in the pancreatic TME. Tumor-associated inflammation involves interactions between various types of immune cells, inflammatory cells, cancer cells, chemokines and cytokines. It has a critical role in initiation, progression, malignant conversion, metastasis and resistance to chemotherapy, radiotherapy or immunotherapy of pancreatic cancer. All the molecules discussed in this review exhibit multiple functions and are derived from immune and non-immune cells. Many of these molecules are also overexpressed by cancer cells as well as cancer-associated stromal cells in the TME. Manipulating cytokine pathways, therefore, represents an effective strategy for pancreatic cancer. However, challenges remain in the translation of basic findings in animal models to treatment in human cancers because of the genetic variability and history of environmental exposures. Moreover, the specific roles of many of these molecules are far from being completely understood. Finally, it is unrealistic to assume that targeting a single cytokine or even a single cell type will yield a satisfactory therapeutic effect. Functionally opposing stromal components have been hypothesized to co-exist in the TME. Single-cell sequencing technologies have been instrumental to define cellular subsets in the TME (134). These technologies can probe cellular and microenvironmental heterogeneity at single-cell resolution, thereby contributing to the improvement of personalized therapeutics. The diversity of CAF populations in pancreatic cancer, including myofibroblastic CAFs, inflammatory CAFs and antigen-presenting CAFs, has been identified by single-cell RNA sequencing (135). The precise roles and plasticity of CAFs need to be investigated, with single-cell studies identifying even greater levels of subtype diversity. Single‐cell sequencing technologies can be used to explore tumor heterogeneity and molecular subtype, the tumor microenvironment and mechanisms associated with progression (136). Moncada et al. identified unique pancreatic cancer cell states and linked these states to the localization of other cell types in the microenvironment. The authors found that subpopulations of macrophages, cancer cells and dendritic cells had distinct co-enrichments with other cell types by applying single-cell RNA sequencing (137, 138). A pancreatic fibroblast lineage that supported anti-tumor immunity was also defined by single-cell analysis. CD105^pos^ pancreatic fibroblasts were tumor permissive, whereas CD105^neg^ fibroblasts supported anti-tumor immunity to control tumor growth (139). Undoubtedly, single-cell sequencing technologies will contribute to new levels of precision and accuracy in cancer research and revolutionize future therapeutic approaches to improve personalized medicine.

In conclusion, better understanding of the crosstalk between inflammation and stromal components in TME is central to understanding the immune tolerance in pancreatic cancer, and future studies elucidating these questions will help towards the development of effective immunotherapies for pancreatic cancer.

## Author Contributions

SL, YL, and YW performed the scientific literature search, collected and analyzed data, designed the review structure and wrote the manuscript. JW, SZ, and HW prepared the tables and figures. All authors have read and approved the final version of the manuscript.

## Funding

The study was supported by the National Natural Science Foundation of China (Grant No.81802888), the Key Technology Research and Development Program of Shandong (No.2018GSF118088), and the General Financial Grant from the China Postdoctoral Science Foundation (No. 2016M592143).

## Conflict of Interest

The authors declare that the research was conducted in the absence of any commercial or financial relationships that could be construed as a potential conflict of interest.

## Publisher’s Note

All claims expressed in this article are solely those of the authors and do not necessarily represent those of their affiliated organizations, or those of the publisher, the editors and the reviewers. Any product that may be evaluated in this article, or claim that may be made by its manufacturer, is not guaranteed or endorsed by the publisher.

## Abbreviations

TME, Tumor microenvironment; IL, interleukin; PSCs, pancreatic stellate cells; CAFs, cancer-associated fibroblasts; TNF-α, tumor necrosis factor α; TAMs, tumor associated macrophages; Tregs, regulatory T cells; MDSCs, myeloid-derived suppressor cells; APCs, antigen-presenting cells; LIF, leukemia inhibitory factor; EMT, epithelial to mesenchymal transition; PI3K, phosphatidylinositol 3-stimulation; PD-L1, programmed death-ligand 1; CTLs, cytotoxic T-lymphocytes; IDO, indoleamine 2,3-dioxygenase; NO, nitric oxide; HMGB1, high mobility group box 1; HIF-1, hypoxia inducible factor-1; VEGF, vascular endothelial growth factor; ET-1, endothelin-1; PDGF, platelet-derived growth factor; PDK, pyruvate dehydrogenase kinase; NRP1, neuropilin 1; FAP, fibroblast activating protein; CSF, colony stimulating factor; MCP-1, monocyte chemotactic protein-1; GPCR, G-protein coupled receptor; IMCS, immature bone marrow cells; GM-CSF, Granulocyte macrophage colony stimulating factor; PGE2, prostaglandin E2; ARG1, arginase 1; iNOS, inducible nitric oxide synthase; NOX2, NADPH oxidase; MHC, major histocompatibility complex; CCL28, chemokine ligand 28; TANs, tumor-associated neutrophils; MCs, mast cells.
